# Validation of the Portuguese version of the Lithium Attitudes Questionnaire (LAQ) in bipolar patients treated with lithium: cross-over study

**DOI:** 10.1186/1745-0179-2-32

**Published:** 2006-11-22

**Authors:** Adriane R Rosa, Ana Cristina Andreazza, Jose Sanchez-Moreno, Fernando K Gazalle, Aida Santin, Airton Stein, Helena MT Barros, Eduard Vieta, Flávio Kapczinski

**Affiliations:** 1 Bipolar Disorders Program, Centro de Pesquisas, Hospital de Clínicas de Porto Alegre, Ramiro Barcelos, 2350, 90035-003, Porto Alegre, RS, Brazil; 2 Department of Biochemistry, Instituto de Ciências Básicas da Saúde, Universidade Federal do Rio Grande do Sul, Ramiro Barcelos, 2600- Anexo, 90035-003, Porto Alegre, RS, Brazil; 3 Departamento de Farmacologia, Fundação Faculdade Federal de Ciências Médicas de Porto Alegre, RS, Brazil; 4 Bipolar Disorders Program, Clinical Institute of Neuroscience, Hospital Clinic de Barcelona, Villarroel 170, Barcelona 08036, Barcelona, Spain; 5 Post-Graduate Psychiatry Program, Universidade Federal do Rio Grande do Sul, Porto Alegre, RS, Brazil

## Abstract

**Background:**

Poor adherence to lithium is very common in bipolar patients and it is a frequent cause of recurrence during prophylactic treatment. Several reports suggest that attitudes of bipolar patients interfere with adherence to lithium. The Lithium Attitudes Questionnaire (LAQ) is a brief questionnaire developed as a means of identifying and grouping the problems patients commonly have with taking lithium regularly. The original version is validated in patients, but a validated version in Portuguese is not yet available.

**Methods:**

One-hundred six patients with bipolar disorder (DSM-IV criteria) criteria under lithium treatment for at least one month were assessed using LAQ. LAQ is a brief questionnaire administered under interview conditions, which includes 19 items rating attitudes towards prophylactic lithium treatment. We analysed the internal consistency, concurrent validity, sensitivity and specificity of the Portuguese version of LAQ.

**Results:**

The internal consistency, evaluated by Cronbach's alpha was 0.78. The mean total LAQ score was 4.1. Concurrent validity was confirmed by a negative correlation between plasma lithium concentration and total LAQ score (r = -0,198; p = 0.048). We analysed the scale's discriminative capacity revealing a sensitivity of 69% and a specificity of 71% in the identification of negative attitudes of bipolar patients.

**Conclusion:**

The psychometric assessment of the Portuguese version of LAQ showed good internal consistency, sensitivity and specificity. The results were similar to the original version in relation to attitudes of bipolar patients towards lithium therapy.

## Background

Bipolar disorder (BD) is a chronic, recurrent illness, affecting 0.3% to 1.5% of the population. BD is associated with important social and economic costs, including loss of productivity, lower quality of life, incremented healthcare costs and suicide [1,2]. Lithium remains as the mainstay in bipolar disorder treatment. It reduces manic symptoms in 73% of the treated patients, twice as many as placebo, and prevents the recurrence of mood episodes [3-6].

Non-adherence rates to long-term prophylactic pharmacotherapy range from 20 to 66% [6-9]. The median duration of lithium adherence is around 76 days [10]. Poor adherence to lithium is, unfortunately, very common and it is the most frequent cause of recurrence during prophylactic treatment [10-14]. Several studies reported that negatives attitudes, such as non-acceptance of lithium effectiveness, opposition to the treatment, denial of the disease and fear of side effects interfere with adherence [15-17].

The 'Lithium Attitudes Questionnaire' (LAQ) was developed as means of identifying and grouping the problems that patients commonly have when taking lithium regularly. The 'LAQ' evaluates the main advantages and disadvantages of lithium treatment. Its subscores are then used to describe patients who expressed opposition to continuing on lithium, and those who missed their hypomanic episodes. The original English version is validated in psychiatric population and has shown adequate reliability [18].

The purpose of the present study was to validate the Portuguese version of LAQ and to describe its psychometric properties. This validation may help the assessment of attitudes that may affect adherence in bipolar patients from Brazil and Portugal.

## Methods

### 2.1. Subjects

The study was conducted in two psychiatric outpatient services specialized in mood disorders in the city of Porto Alegre, Brazil. The psychiatric outpatients selected were diagnosed with bipolar disorder according to DSM-IV criteria.

Patients were evaluated for both their symptoms and general state in weekly consultations. Patients participated in psycho-educational groups about the use of lithium and support groups to discuss topics related to the disorder. After giving the informed consent, patients were interviewed and had a lithium blood level assessment scheduled.

The study was approved by the Ethics Committee of the Hospital Materno Infantil Presidente Vargas and Hospital de Clínicas de Porto Alegre, where the research took place and was carried out in compliance with the Helsinki Declaration.

### 2.2. Variables

#### Demographical data

Every subject gave information about marital status, work, age, gender and level of education.

#### Current clinical status

Bipolar patients were diagnosed using a Structured Clinical Interview (SCID) and all patients were followed up prospectively using mood charts. The clinical status was also assessed on the day of the LAQ application and blood collection for lithium measurements.

#### LAQ

LAQ is a brief questionnaire comprising 19 items which rate attitudes towards prophylactic lithium treatment. Seven subscales assess the resistance to prophylaxis in general (LAQ 1), denial of therapeutic effectiveness of lithium as a prophylactic agent (LAQ 2), fear of side effects (LAQ 3), difficulties with the daily routine medication intake (LAQ 4), denial of the severity of the illness (LAQ 5), negative attitudes toward drugs in general (LAQ 6) and lack of information about lithium prophylaxis (LAQ 7) [16,18]. The correct answers are: 1-N; 2-Y; 3-N; 4-N; 5-Y; 6-N; 7-Y; 8-N; 9-Y; 10-N; 11-Y; 12-N; 13-Y; 14-N; 15-Y; 16-N; 17-Y; 18-Y; 19-N. The items are posed using a Yes/No format with low scores indicating positive attitudes and high scores indicating negative attitudes The total LAQ score is obtained by adding together the responses to the 19 points. If a patient obtains a score > 4 this indicates a negative attitude versus lithium treatment, as shown in Table 1.

**Table 1:** Questionário de Atitudes em relação ao Lítio (LAQ)

Marque com um X as respostas que o Sr considerar correta.	
1. O Sr(a). acha perfeitamente aceitável tomar LÍTIO por vários anos?	(1)S (2) N
2. O Sr(a). toma LÍTIO somente quando sente necessidade?	(1)S (2) N
3. O Sr(a) acha que vale a pena tomar LÍTIO, apesar dos efeitos colaterais?	(1)S (2) N
4. Tomar LÍTIO conforme receitado pelo seu médico é fácil no seu dia-dia?	(1)S (2) N
5. É melhor aliviar o estresse, do que tomar LÍTIO para ficar bem (estável)?	(1)S (2) N
6. Sr(a). considera que a LÍTIO é uma necessidade atual para o seu bem-estar?	(1)S (2) N
7. Sr(a) se preocupa com os efeitos colaterais do LÍTIO mesmo quando sente-se bem?	(1)S (2) N
8. A maioria das pessoas que o Sr(a) conhece acham necessário que tome o LÍTIO?	(1)S (2) N
9. Sr(a). ás vezes tenta esquecer que está doente, e por isso para de tomar os seus comprimidos de LÍTIO?	(1)S (2) N
10. Sr(a). confia tanto nos seus comprimidos de LÍTIO, que se por algum motivo fosse interrompido o seu tratamento, o Sr(a) ficaria preocupado?	(1)S (2) N
11. As pessoas precisam lhe lembrar de tomar o LÍTIO?	(1)S (2) N
12. Sr(a). aceita bem o LÍTIO, mesmo sabendo que é necessário fazer exames de sangue e check up regulares?	(1)S (2) N
13. Sr(a). ás vezes pensa que o LÍTIO é uma maneira artificial de lhe manter bem, e que deveria conseguir viver sem ela?	(1)S (2) N
14. É fácil lembrar as horas certas de tomar LÍTIO?	(1)S (2) N
15. Sr(a). freqüentemente duvida que a sua condição de saúde seja tão seria, que justifique o uso do lítio por vários anos?	(1)S (2) N
16. Sr(a). tem um conhecimento adequado sobre os efeitos da LÍTIO?	(1)S (2) N
17. Se o Sr(a) ficasse bem por vários meses, deixaria de tomar a LÍTIO?	(1)S (2) N
18. Se a sua rotina diária mudar por alguma razão, o Sr(a). terá dificuldade de tomar seus comprimidos de LÍTIO?	(1)S (2) N
19. Sr(a). está convencido dos efeitos benéficos do LÍTIO baseado na sua própria experiência?	(1)S (2) N

The adaptation of LAQ was carried out using an instrument translated into Portuguese after translation/back translation procedures [19,20]. The items resulting in optimal word equivalence with the original text were analysed and discussed by five psychiatric investigators who agreed with the final version. After these procedures, investigators who were fluent in both English and Portuguese evaluated the degree of equivalence between the original English and the Portuguese version. Finally, the comprehension of each item was assessed with a sample of 106 bipolar patients. The Portuguese version of the LAQ used in this study was found to be appropriate and correctly understood by psychiatric patients.

The LAQ was administered on one occasion and the time spent in the application of LAQ was 6–8 minutes. On this occasion venous blood was drawn from each patient to assess levels of lithium. The patients did not take lithium on the morning of the interview.

We analysed the internal consistency, concurrent validity in relation to plasma lithium concentration, sensitivity and specificity. The psychometric characteristics of the LAQ are derived from the administration of the questionnaire, including all the subjects who completed the analysis. Internal consistency was assessed by Cronbach's Alpha for the total scale and each individual item. Concurrent validity was studied considering plasma lithium concentration and the score obtained using LAQ. Pearson coefficient was used for verify correlation between LAQ scores and plasma lithium concentration. The cutt-off used was 4 (from the original reference).

To study the sensitivity, specificity and positive and negative predicative of the LAQ, we have used the proportion of adherent bipolar patients as compared to plasma lithium concentration. A discriminate analysis was carried out using data obtained by means of LAQ application.

#### Lithium plasma concentration

The subjects were instructed to be at the hospital early in the morning, without having taken their lithium dose, respecting a 12-hour interval between the last dose of lithium and the blood sampling. Venous blood was collected from each patient into Vacutainer tube containing edetic acid. The whole blood was then centrifuged 1600 × g for 10 minutes and the plasma removed by aspiration. A 1/20 dilution in water was made of 99 μl of plasma. Lithium concentrations were measured in plasma dilutions by the indirect method, using an Instrumentation Laboratory CELM Flame Photometer. Assays were performed in duplicate [21].

### Statistical analysis

Statistical analysis was performed using SPSS for Windows – Version 12.0 (SPSS Inc., Chicago, IL, USa). The Kolmogorov-Smirnov test was used to compare the observed cumulative distribution to a theoretical cumulative distribution function. ANOVA test was used for compare LAQ scores and plasma levels of lithium. Pearson's correlation coefficient was performed to examine the relationship between plasma lithium concentration and LAQ scores. The binomial variables (clinical state and LAQ scores) were compared using Chi-Square test.

## Results

The sample included 73 (68.9%) women, with a mean age 43.5 ± 9.8 and 33 men with a mean age of 41.6 ± 9.7. At the time when the present study was carried out, there were 77 (71.7%) euthymic patients, 12 (11.1%) manic and 17 (17,2%) depressed, according to the mood charts and physician is assessment. 40 (37.7%) of patients were smokers, 70 (66%) used coffee on a regular basis, and 68 (64.2%) used tea on a regular basis.

The answers to the LAQ were obtained from 106 bipolar patients and the mean score on the LAQ TOTAL was 4.1 ± 0.5 for men and 4.1 ± 0.4 for women. Therefore, gender did not seem to influence any of the adherence parameters evaluated using LAQ scale or lithium plasma. The demographics and clinical characteristics are shown in Table 2.

**Table 2:** Demographics and Scores for lithium treatment parameters in 106 bipolar patients

	Average	SD
Age	42,9	9,79
Education level	9,2	3,4
Plasma lithium	0,86	0,25
LAQ TOTAL	4,1	3,37
LAQ1	0,96	1,16
LAQ2	0,13	0,39
LAQ3	0,57	0,6
LAQ4	0,57	0,89
LAQ5	0,67	0,77
LAQ6	0,78	0,82
LAQ7	0,42	0,5

All LAQ items were answered by patients in every test session. No patient posed objections to completing the questionnaire. The internal consistency coefficient was obtained for the 19 items as shown Table 3 and the mean Cronbach's alpha of 0.78 for the total scale indicates that the items are sufficiently homogeneous.

**Table 3:** Internal Consistency Reliability

	Scale Mean	Scale Variance	Corrected item	Alpha
Question 1	3,7453	9,4107	0,5778	0,7543
Question 2	4,0283	10,904	0,2149	0,7803
Question 3	4	10,2667	0,5044	0,7654
Question 4	4,0094	10,4666	0,42	0,7703
Question 5	3,9717	10,4659	0,3496	0,7732
Question 6	4,066	10,9385	0,2968	0,7779
Question 7	3,6415	10,5179	0,1792	0,7882
Question 8	3,8679	10,8014	0,1311	0,7886
Question 9	3,8585	10,1417	0,3713	0,7715
Question 10	3,8386	9,6788	0,5355	0,7588
Question 11	3,9245	10,0704	0,4628	0,7654
Question 12	3,9811	10,3806	0,4058	0,7701
Question 13	3,6887	9,6069	0,4884	0,7619
Question 14	3,9434	10,1491	0,4538	0,7664
Question 15	3,7547	10,0726	0,3451	0,774
Question 16	3,6792	10,9247	0,547	0,7979
Question 17	3,8868	10,0442	0,4325	0,7671
Question 18	3,9717	10,7135	0,2343	0,7798
Question 19	4,0094	10,3523	0,4828	0,7671

The internal consistency coefficient (Cronbach's alpha) obtained was 0.78 for the total scale and was superior to 0.7 for each of the 24 items, indicating sufficiently homogeneous.

We analysed the scale's discriminative capacity for bipolar patients by means of the performance of 'attitudes toward lithium treatment' on sensitivity and specificity analysis. The calculated sensitivity and specificity were 69% and 71%, respectively.

**Figure 1:**
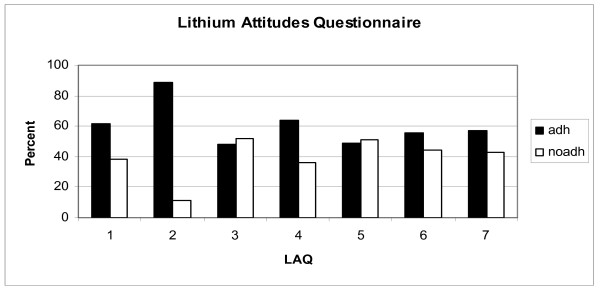
Percent of adherent (adh) and nonadherent (noadh) patients scoring positively for each LAQ subscore. (1) resistance to prophylaxis in general; (2) denial of therapeutic effectiveness of lithium as a prophylactic agent; (3) fear of side effects (LAQ 3); (4) difficulties with the daily routine medication intake; (5) denial of the severity of the illness; (6) negative attitudes toward drugs in general; (7) lack of information about lithium prophylaxis (LAQ 7)

Concurrent validity was assessed using plasma lithium concentration and showed a significant negative correlation between the total LAQ score and lithium levels (r = -0.198; p = 0.048). The number of patients scoring positively for each LAQ subscore during the test completion is shown in Figure 1.

Figure 1 shows that LAQ's subscore 3, regarding the side effects of lithium, was considered a disadvantage of treatment by the majority of respondents. Patients also reported subscore 5 (denial of illness severity) as a source of noncompliance with Li prophylaxis. Subcultural attitudes, as assessed using subscore 6, was considered as a disadvantage in 44.4% of the positive respondents and dissatisfaction with factual knowledge of Li (subscore 7) was rated as a disadvantage in 42,5% of the positive respondents.

We observed statistically significant differences between the LAQ 1 and scores of patients currently manic or euthymic and significant differences between the LAQ 2 and scores of patients currently manic, depressed or euthymic (Pearson Chi-Square < .05) (see Table 4). This result indicates that acute symptoms interfered in lithium treatment adherence.

**Table 4:** Clinical state an scores of LAQ

	LAQ 1		P	LAQ 2		p
Clinical state	Adherent	no adherent		adherent	no adherent	
Euthymic	52 (80%)	23 (57,5%)	0,011	73 (77,7%)	3 (25%)	0,001
Manic	3 (4,6%)	9 (22,5%)	0,012	8 (8,5%0	4 (33,3%)	0,001
Depressive	10 (15,4%)	8 (20%)	0,081	13 (13,8%)	5 (41,7%)	0,001

A Pearson Chi-Square showed significant differences between the LAQ 1 scores of patients currently euthymic or manic. There were also significant differences between the LAQ 2 scores of patients currently euthymic, manic or depressive.

## Discussion

Unfortunately, poor adherence to lithium is very common and previous studies have already pointed out that it is the most frequent cause of recurrence during prophylactic lithium treatment [13,14,22]. Therefore, it is reasonable try and identify potential reasons which lead patients to discontinue lithium treatment. The Portuguese version of the LAQ scale may become particularly useful because Portuguese is a language which is spoken by a population of 202 million people, including Brazil and Portugal. Hence, it is reassuring to find that the psychometric assessment of the Portuguese version of LAQ showed good sensitivity and specificity. In a sample of bipolar patients the Portuguese version of the LAQ used the cut-off point of 4, which mirrors the English original version; this indicates that four or more points at LAQ reflect the existence of negative attitudes towards lithium treatment.

The LAQ scale showed very good internal consistency and all items presented a Cronbach's alpha above 0.7, as internationally accepted. In the convergent validity analysis we observed a negative correlation between lithium plasma levels and total scores of LAQ. This was due to the fact that, in non-adherent patients (plasma lithium levels < 0.6 mmol L or > 1.2 mmol L), negative attitudes about lithium treatment were greater than those observed among adherent patients.

The analysis of each LAQ subscores showed that subscore 3, regarding side effects of lithium, was considered an important disadvantage of treatment by the majority of respondents, which is in line with the original study. This result is important because fear of side effects, and not the side effects themselves seem to pose important barriers for the use of lithium. Indeed, Scott and Pope [15] reported that many patients with affective disorders feared harmful effects of the use of lithium in the long run. Patients also reported that denial of the severity of the illness, negative attitudes toward drugs in general and lack of information about lithium prophylaxis were also identified as caveats of lithium therapy. These results are in accordance with previous studies which highlighted the fact that non-adherent bipolar patients tend to present negative attitudes towards lithium treatment [14-17,23].

Further, the scale proved to be sensitive to mood changes, as differential scores were captured during depression, mania and euthymia. Euthymic patients showed more positive attitudes than manic and depressed patients. The scores obtained in items such as LAQ 1 (resistance to prophylaxis in general) and LAQ 2 (denial of therapeutic effectiveness of lithium as a prophylactic agent) were the main differences between euthymic patients and those who were either manic or depressed. These results were reported in the original English version, where hypomanic patients showed more resistance to prophylaxis in general [24]. Negative attitudes strengthen the hypothesis that attitudes towards treatment play a major role in the effectiveness of lithium long-term prophylaxis.

Several studies [22,24] found a possible effect of education in improving adherence. It is reasonable to suppose that this effect is in some extent mediated via patient's aquisition of a positive attitude towards lithium treatment [16]. LAQ is a rapid, reliable screening instrument which presents a good level of acceptability by patients. The subcategories of items, from which the LAQ subscores derive, describe specific problem areas, most of which are considered by patients to represent noteworthy disadvantages of lithium therapy [18]. The importance of this finding is that all factors can be assessed routinely in day-to-day clinical practice, with no need of long questionnaires. The identification of negative attitudes toward lithium treatment may be an important tool in order to increase adherence and consequently reduce the number of relapses, economic burden and rates of mortality associated with BD.

It is important to highlight the fact that individuals who are non-adherent to lithium are also likely to fail to agree to participate or fail to adhere with research protocols. Another point to be considered is that we did not assess current symptom severity and comorbidities. It is also important to mention that this was a crossover study, as we assessed attitudes about the use of medication in only one occasion. As yet, there is no research on the stability or variability of such beliefs over time.

## Conclusion

In conclusion, the LAQ scale is a rapid and reliable screening instrument which may help to identify negative attitudes towards lithium treatment. The present study showed that the Portuguese version is valid for the assessment of bipolar patients in Portuguese-speaking countries.

## Competing interests

The author(s) declare that they have no competing interests.
